# Impact of National Containment Measures on Decelerating the Increase in Daily New Cases of COVID-19 in 54 Countries and 4 Epicenters of the Pandemic: Comparative Observational Study

**DOI:** 10.2196/19904

**Published:** 2020-07-22

**Authors:** Carlos K H Wong, Janet Y H Wong, Eric H M Tang, Chi Ho Au, Kristy T K Lau, Abraham K C Wai

**Affiliations:** 1 Department of Family Medicine and Primary Care Li Ka Shing Faculty of Medicine The University of Hong Kong Hong Kong China (Hong Kong); 2 School of Nursing Li Ka Shing Faculty of Medicine The University of Hong Kong Hong Kong China (Hong Kong); 3 Emergency Medicine Unit Li Ka Shing Faculty of Medicine The University of Hong Kong Hong Kong China (Hong Kong)

**Keywords:** COVID-19, national containment, lockdown, curfew, stay-at-home, epidemic curve

## Abstract

**Background:**

Coronavirus disease (COVID-19) is a worldwide epidemic, and various countries have responded with different containment measures to reduce disease transmission, including stay-at-home orders, curfews, and lockdowns. Comparative studies have not yet been conducted to investigate the impact of these containment measures; these studies are needed to facilitate public health policy-making across countries.

**Objective:**

The aim of this study was to describe and evaluate the impact of national containment measures and policies (stay-at-home orders, curfews, and lockdowns) on decelerating the increase in daily new cases of COVID-19 in 54 countries and 4 epicenters of the pandemic in different jurisdictions worldwide.

**Methods:**

We reviewed the effective dates of the national containment measures (stay-at-home order, curfew, or lockdown) of 54 countries and 4 epicenters of the COVID-19 pandemic (Wuhan, New York State, Lombardy, and Madrid), and we searched cumulative numbers of confirmed COVID-19 cases and daily new cases provided by health authorities. Data were drawn from an open, crowdsourced, daily-updated COVID-19 data set provided by Our World in Data. We examined the trends in the percent increase in daily new cases from 7 days before to 30 days after the dates on which containment measures went into effect by continent, World Bank income classification, type of containment measures, effective date of containment measures, and number of confirmed cases on the effective date of the containment measures.

**Results:**

We included 122,366 patients with confirmed COVID-19 infection from 54 countries and 24,071 patients from 4 epicenters on the effective dates on which stay-at-home orders, curfews, or lockdowns were implemented between January 23 and April 11, 2020. Stay-at-home, curfew, and lockdown measures commonly commenced in countries with approximately 30%, 20%, or 10% increases in daily new cases. All three measures were found to lower the percent increase in daily new cases to <5 within one month. Among the countries studied, 20% had an average percent increase in daily new cases of 30-49 over the seven days prior to the commencement of containment measures; the percent increase in daily new cases in these countries was curbed to 10 and 5 a maximum of 15 days and 23 days after the implementation of containment measures, respectively.

**Conclusions:**

Different national containment measures were associated with a decrease in daily new cases of confirmed COVID-19 infection. Stay-at-home orders, curfews, and lockdowns curbed the percent increase in daily new cases to <5 within a month. Resurgence in cases within one month was observed in some South American countries.

## Introduction

The coronavirus disease (COVID-19) epidemic is caused by severe acute respiratory syndrome coronavirus 2 (SARS-CoV-2); it is the third coronavirus-associated epidemic to emerge from a species leap from wild animals to humans, after severe acute respiratory syndrome (SARS) in 2003 and Middle East respiratory syndrome (MERS) in 2012 [1,2]. Coronavirus infection causes acute respiratory illness that is usually self-limiting but can be severe in some cases [3]. Coronaviruses primarily infect the upper respiratory and gastrointestinal tracts of birds and mammals. However, once a human is infected, they can transmit the coronavirus to other humans through respiratory droplets and aerosols from coughing and sneezing, like other respiratory pathogens [4]. In Wuhan, China, it was estimated that the basic reproductive number for SARS-CoV-2 was 2.68 (95% CI 2.47-2.86) and its doubling time was 6.4 days (95% CI 5.8-7.1) [5] in the early phase of the pandemic. Since January 2020, following the lockdown of Wuhan, increasing numbers of SARS-CoV-2–infected cases have been reported outside the city [6]. The COVID-19 pandemic began with small chains of transmission in China and nearby cities, which then became large chains of extensive spread in countries worldwide. As of July 2, 2020, over 10 million confirmed cases and more than 510,000 deaths have been recorded globally [7]. National responses of containment measures, such as stay-at-home orders, curfews, and lockdowns, have varied across countries with different characteristics. To our knowledge, there is no comparative study investigating the impact of these containment measures on COVID-19 transmission in countries with respect to geographical location, income status, containment measures imposed, and early and late responses to the COVID-19 pandemic.

From the perspective of public health, principles of controlling contagious disease transmission focus mainly on early detection via testing and contact tracing, in addition to prevention of transmission by containment measures such as stay-at-home orders, curfews, lockdowns, quarantine of exposed individuals [8], and travel or trade restrictions [9]. Additionally, behavioral interventions such as personal protective measures (such as hand hygiene and respiratory etiquette) and social or physical distancing measures (such as isolation of sick individuals, school measures and closures, workplace measures and closures, and avoiding crowding) should be advocated at the community level to facilitate the flattening of the epidemic curve [10].

Quarantine has been used as an effective tool to control communicable disease outbreaks throughout history [11]. The quarantine period provides ample time for the incubation period to complete; therefore, asymptomatic cases will become symptomatic and can therefore be identified. Quarantine is most successful in settings where there is prompt detection of new cases, where contacts can be listed and traced within a short time frame, followed by a prompt issuance of quarantine with voluntary compliance. This may not be applicable to the case of COVID-19 infection because the science and epidemiology of the disease are still largely unknown. At the time of preparation of this paper, in a rapid review assessing the effects of quarantine (alone or in combination with other measures) of individuals who had contact with confirmed cases of COVID-19 infection, who travelled from countries with a declared outbreak, or who lived in regions with high transmission of the disease, it was concluded that insufficient evidence was available [12].

Initial analysis of data from China collected in February 2020 suggested that the epidemic did not expand exponentially. Public response to the epidemic in addition to containment policies was found to be effective despite the initial increase in the number of confirmed cases [13]. The initial transmission rate (R_0_) was reduced from 2.6 to 0.62 (95% CI 0.37-0.89) due to the stay-at-home order in the United Kingdom [14]. Similar changes in effective transmission rate (R_t_) were observed in the United States and many European countries after population-level containment interventions were implemented [15,16]. However, worldwide evidence to guide policy makers on effective control of the pandemic is still lacking.

Nonpharmaceutical public health measures at the individual level (physical distancing, use of face masks, and wearing of eye protection to reduce person-to-person transmission) have been studied by the COVID-19 Systematic Urgent Review Group Effort (SURGE) [17]. However, the effectiveness of measures implemented at the national level is a knowledge gap that should be filled. In this study, we aimed to describe and evaluate the impact of national containment measures and policies (stay-at-home orders, curfews, and lockdowns) on decelerating the increase in daily new cases of COVID-19 infection in 54 countries and 4 epicenters of the pandemic in different jurisdictions worldwide.

## Methods

### Data Source

We used an open, crowdsourced, daily-updated COVID-19 data set provided by Our World in Data [18]. This public domain repository provides numbers of cumulative confirmed cases, confirmed cases per 1 million people, confirmed daily new cases, cumulative deaths, and daily deaths for each country associated with the European Centre for Disease Prevention and Control and the total number of tests for COVID-19 performed per 1000 people as reported by the health authority of each country. Because the most common test for COVID-19 is the polymerase chain reaction (PCR) test, our data source tracked the number of tests for COVID-19 in terms of the number of PCR tests performed or the number of individuals who were tested for COVID-19 reported by the health authority of each country. Cumulative confirmed cases and daily new cases in four epicenters (Wuhan, the Lombardy region of Italy, New York State, and Madrid) were obtained from the websites of national health authorities. Commencement dates of countrywide containment measures were gathered from the national health ministries and health authorities by authors CW and EHMT and were cross-checked by the last author (AW). Characteristics including the continent, income level classified by the World Bank [19], types of containment measures (stay-at-home order, curfew, or lockdown), effective dates of containment measures, and numbers of confirmed cases on the effective dates of containment measures were retrieved for each country and epicenter. All crowdsourced data were available up to June 20, 2020.

Regarding the three containment measures under investigation, a stay-at-home order was defined as limited outdoor movement except essential activity, curfew was defined as a stay-at-home order during specific time periods, and lockdown was defined as restriction of population mobility within a specific region or country. Acknowledging that variations in the adoption of containment measures may exist across regions or countries, the main difference between a stay-at-home order and a lockdown was denoted as the restriction of people moving in or out of a region being imposed with the latter but not the former. Accordingly, the containment measure introduced in the United States would be classified as a stay-at-home order.

### Ethics Approval

This study used open-sourced, secondary dataset, and was exempted from ethics review of Institution Review Board.

### Outcome Definitions

The primary outcome of this study was the percent increase in daily new cases from 7 days prior (day –7) to 30 days after (day +30) the commencement of measures in countries and epicenters. This specific timeframe between day –7 and day +30 ensured that complete daily new case data were obtained for all included countries and epicenters. The percent increase in daily new cases on day *t* was defined as the daily new cases on day *t* divided by the cumulative confirmed cases on day *t* – 1, where *t* was between –7 and 30. When estimating the percent increase in daily new cases by characteristic group, estimates were calculated as the total daily new cases in the locations within the group on day *t* divided by the total cumulative confirmed cases of the locations within the group on day *t* – 1. The denominator and numerator in the fraction were two summations; the summations were taken over the countries with nonmissing numbers of cases on days *t* and *t* – 1, respectively. This percent increase can be represented by the weighted sum of the percent increase of the component locations, where the weight is calculated based on the cumulative cases in the country on day *t* – 1:


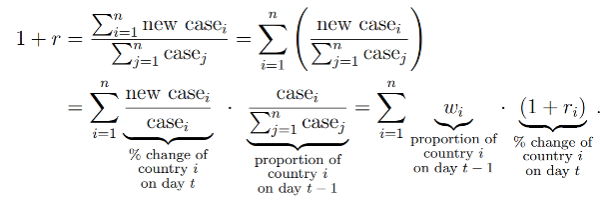


Average increase in daily new cases from day-7 to day-1 was calculated by method of geometric mean.

### Data Analysis

We used line graphs to represent the trends in the percent increase in daily new cases for each type of containment measure and by the measure start date, continent of the country or epicenter, income level, and average percent increase in daily new cases before the start of containment measures. The formula proposed by Waller et al [20] was used to estimate the 95% CI for the weighted proportion. The 95% CI for the percent increase of a location was estimated by the method of the binomial proportion confidence interval, while that for a group was estimated by the method of the weighted binomial proportion confidence interval. The calculation of the percent increase in daily new cases at time *t* was excluded in a particular group and country if the daily new cases data were missing at time *t* – 1 and time *t*. Scatter plots were used to visualize the percent increase in daily new cases at day +7, day +14, day +21 and day +30 for each country or epicenter by the average percent increase in cases before the commencement of the containment measures and after the start dates of the containment measures. The relationship between the number of diagnosed cases and the number of tests performed for each location was also assessed using line graphs.

All statistical analyses and figure generations were performed using Stata version 16.0 (StataCorp LLC).

## Results

This study included 122,366 patients with confirmed COVID-19 infection from 54 countries and 24,071 patients from 4 epicenters on the days when stay-at-home orders, curfews, or lockdowns were implemented between January 23 and April 11, 2020. The containment measures at the national level are summarized in Multimedia Appendix 1. Of the 54 countries, 31 (57%) adopted lockdowns, 17 (32%) adopted curfews, and 6 (11%) adopted stay-at-home orders. All countries initiated their containment policies after March 9, 2020, except for China (January 23, 2020).

**Figure 1:**
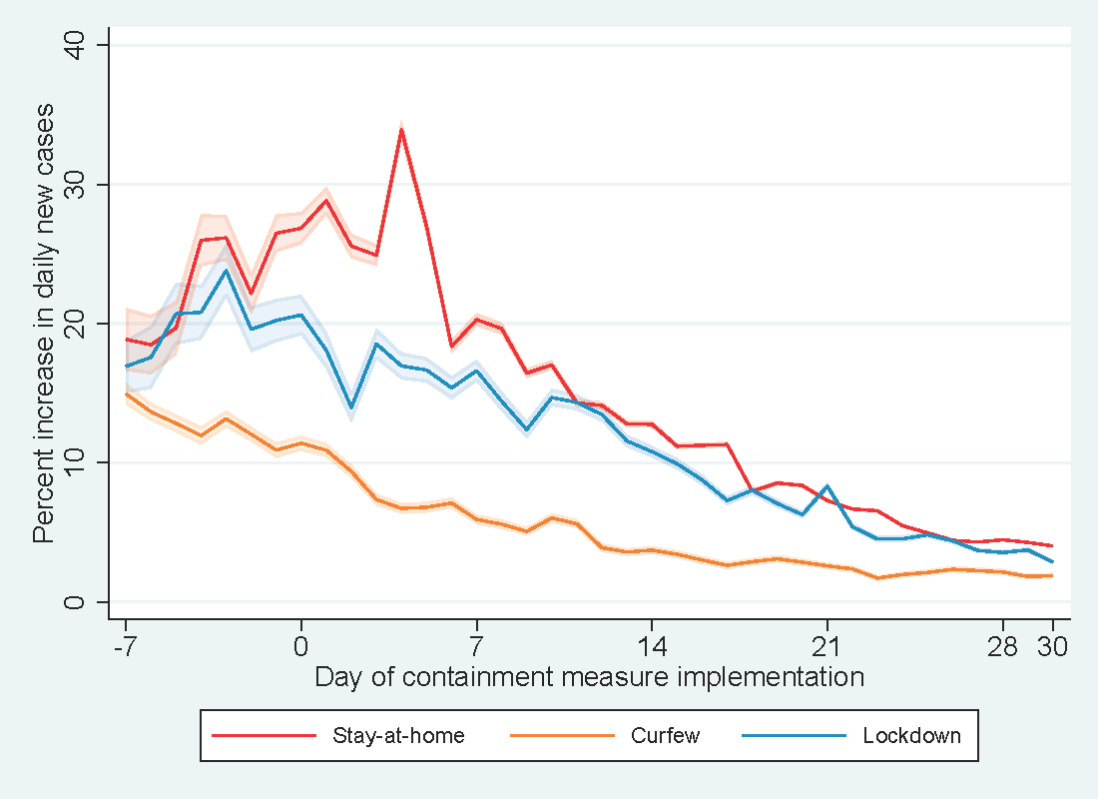
Percent increase in daily new cases vs days since containment measure by type of containment measure.

**Figure 2:**
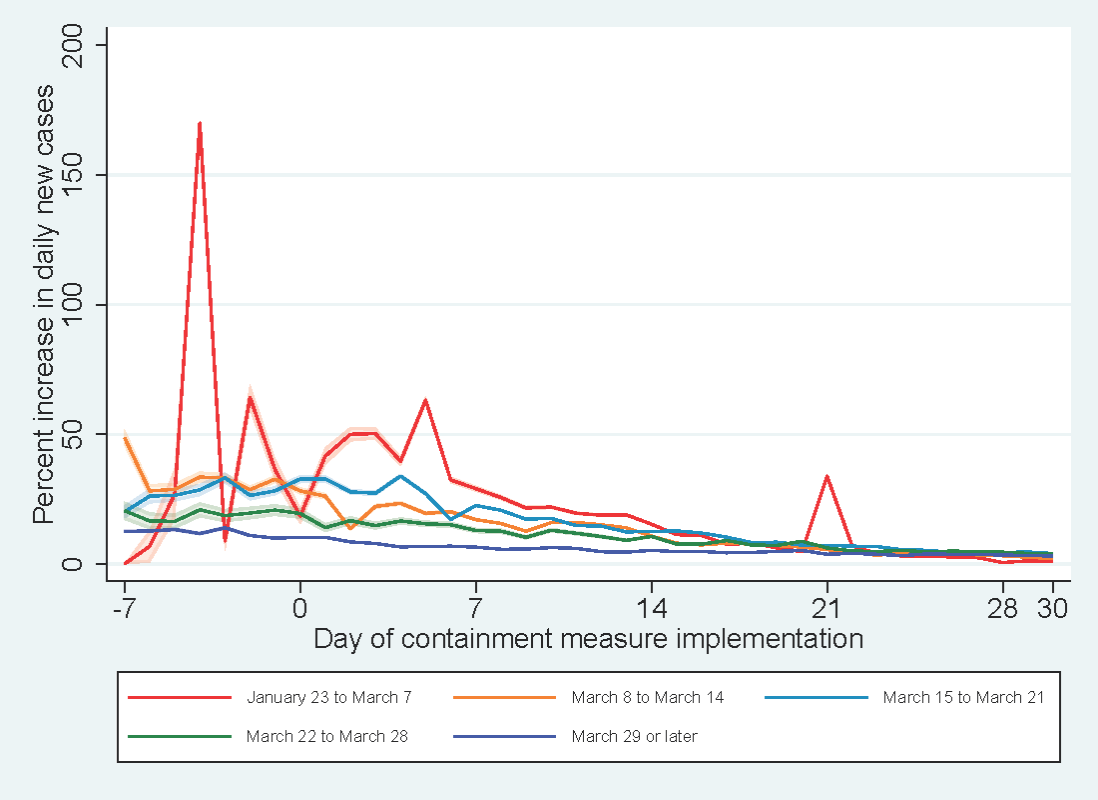
Percent increases in daily new cases vs days since implementation of containment measures by the start date of the containment measure.

Regarding the type of containment measure, countries adopting stay-at-home orders, curfew, and lockdown demonstrated a decreasing trend in percent increase in daily new cases of COVID-19 infection after the commencement of the measures (Figure 1). In countries with stay-at-home orders, curfews, or lockdowns, the percent increase was observed to be curbed to <5 within one month. The percent increase in daily new cases decreased from 26.9 (95% CI 25.7%-28.0%) at baseline to 20.3 (95% CI 19.8%-20.7%) at day +7, 12.8 (95% CI 12.6%-13.0%) at day +14, 7.29 (95% CI 7.17%-7.41%) at day +21, and 4.03 (95% CI 3.96%-4.10%) at day +30 for countries introducing stay-at-home orders. For countries introducing curfew, the percent increase in daily new cases decreased from 11.4 (95% CI 10.9-11.9) at baseline to 5.93 (95% CI 5.61-6.26) at day +7, 3.73 (95% CI 3.47-3.98) at day +14, 2.60 (95% CI 2.38-2.81) at day +21, and 1.89 (95% CI 1.71-2.07) at day +30. Meanwhile, the percent increase in daily new cases decreased from 20.6 (95% CI 19.2-22.1) at baseline to 16.6 (95% CI 15.9-17.4) at day +7, 10.8 (95% CI 10.4-11.2) at day +14, 8.32 (95% CI 8.06-8.57) at day +21 and 2.88 (95% CI 2.73-3.02) at day +30 for countries introducing lockdown. For the start date of the intervention (Figure 2), a persistent drop in the percent increase in daily new cases was also observed at the initial stage of the containment measures, except in China.

**Figure 3:**
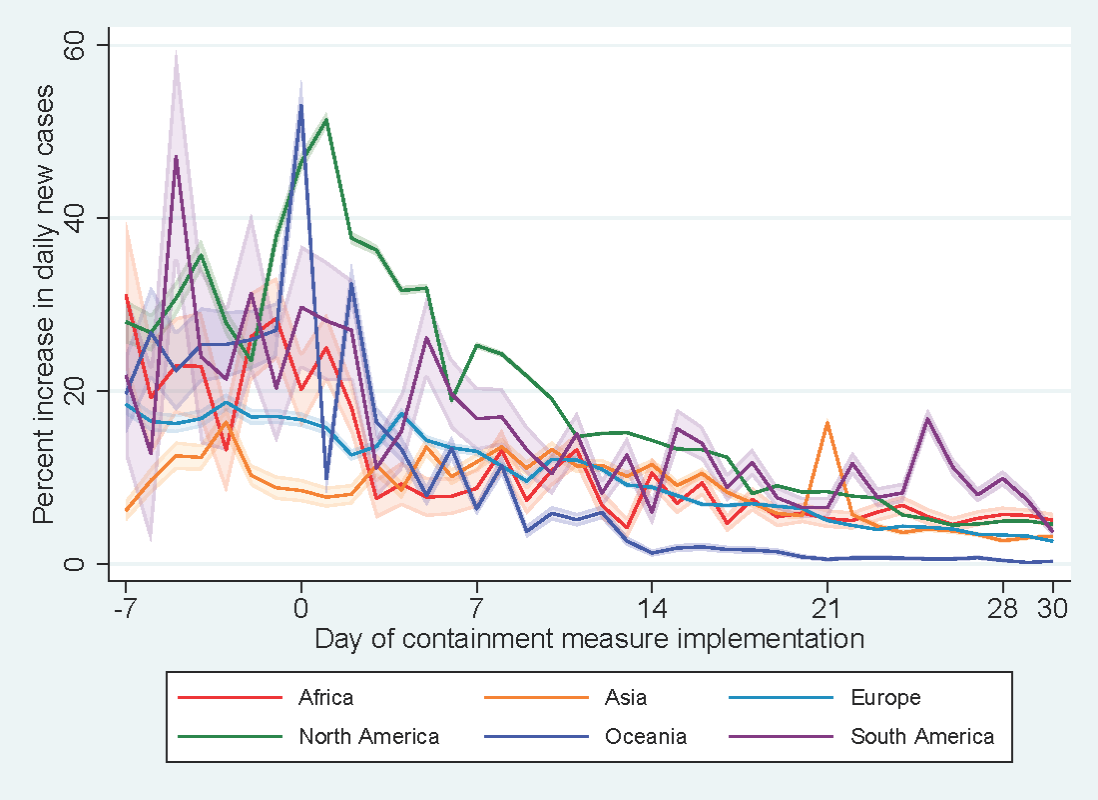
Percent increases in daily new cases against days since implementation of containment measures by continent.

**Figure 4:**
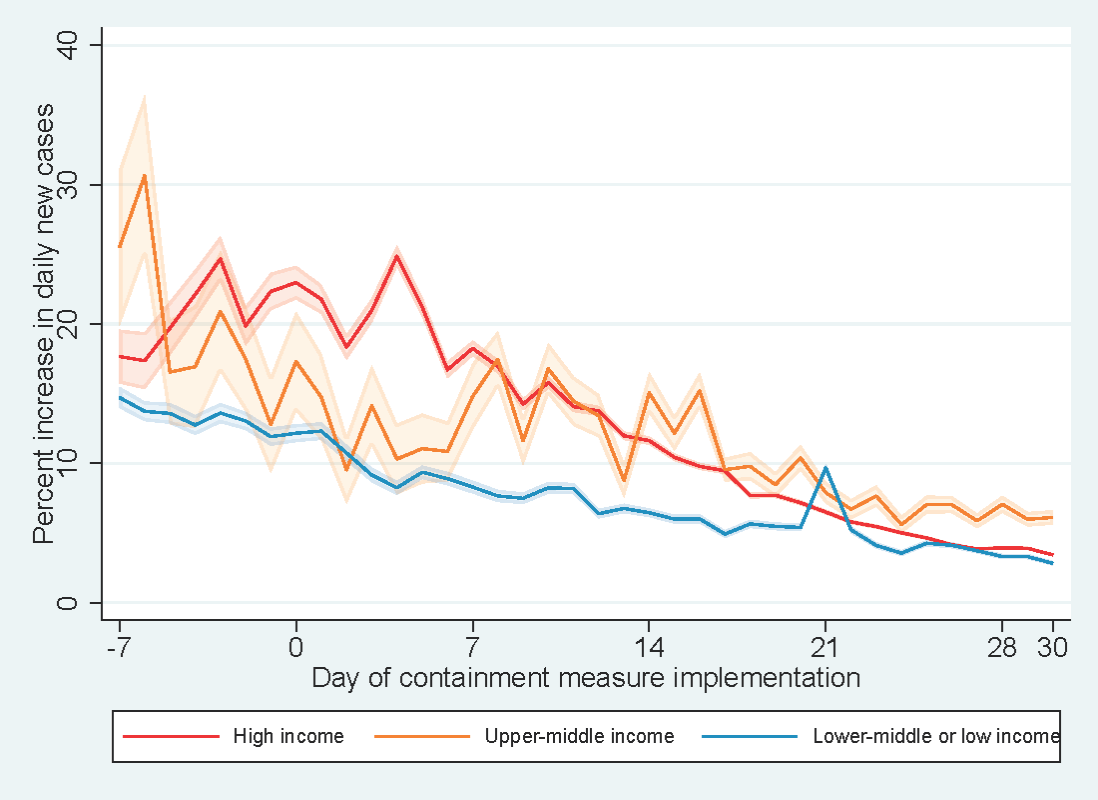
Percent increases in daily new cases vs days since implementation of containment measures by income level.

Within seven days of commencement of the containment measures, a decreasing trend in the percent increase in daily new cases was observed across continents (Figure 3). The introduction of a new COVID-19 case classification in China at day +21 led to a spike in the percent increase in daily new cases in Asia. Resurgence in COVID-19 cases in South American countries was observed with a spike in percent increase in daily new cases at day +25, more than two weeks after the implementation of containment measures. By income level (Figure 4), a decreasing trend in the percent increase in daily new cases was observed for high-income countries since the initiation of containment measures.

**Figure 5:**
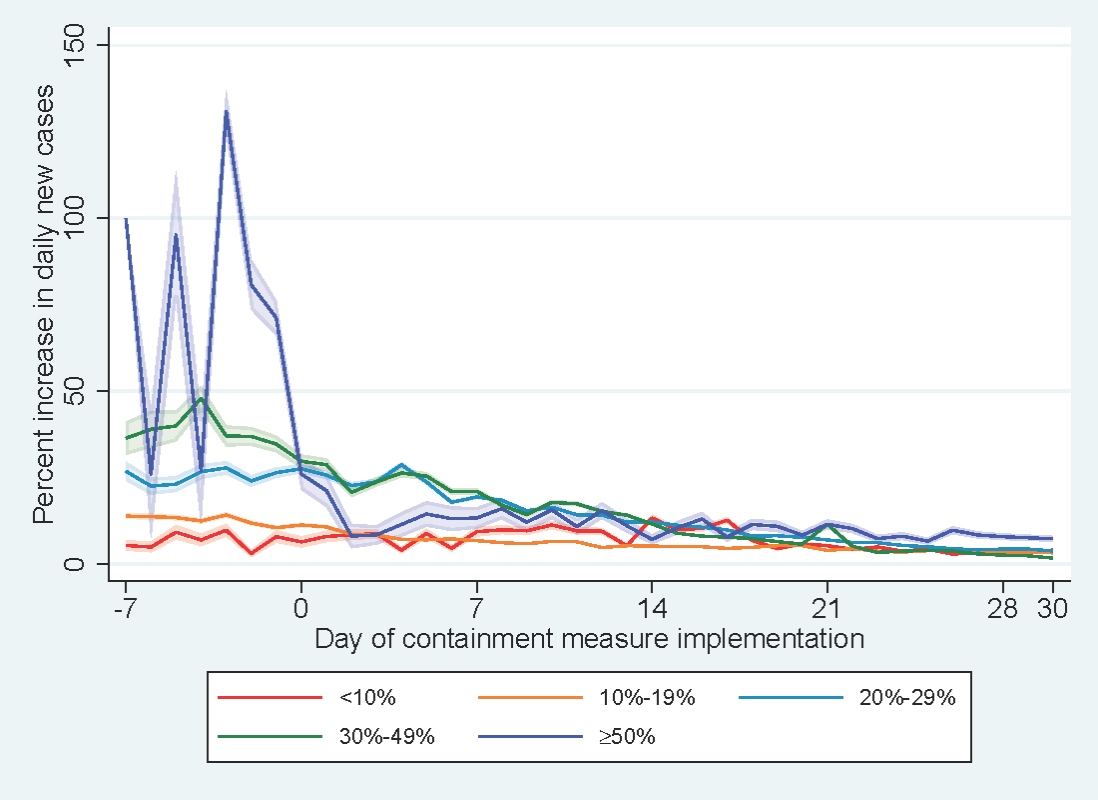
Percent increases in daily new cases vs days since implementation of containment measures by average percent increase in daily new cases before the containment measures.

**Figure 6:**
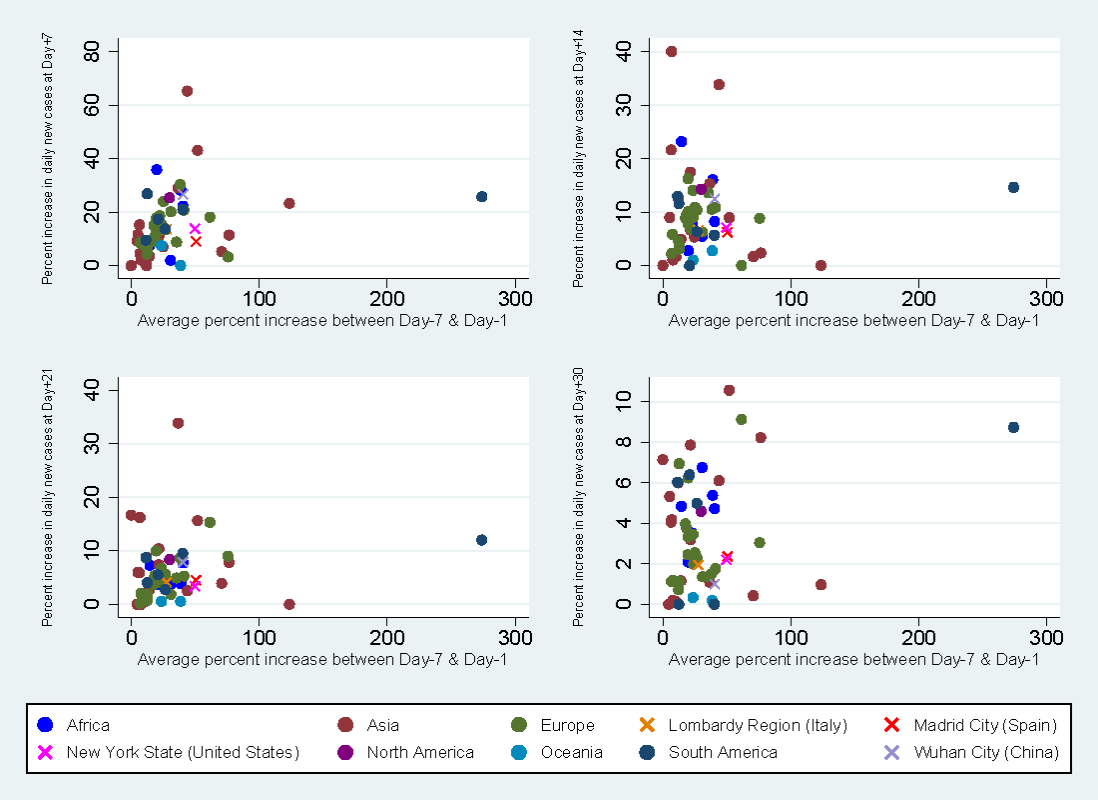
Percent increases in daily new cases at day +7 (A), day +14 (B), day +21 (C) and day +30 (D) against average percent increase in daily new cases before intervention by country and epicenter.

**Figure 7:**
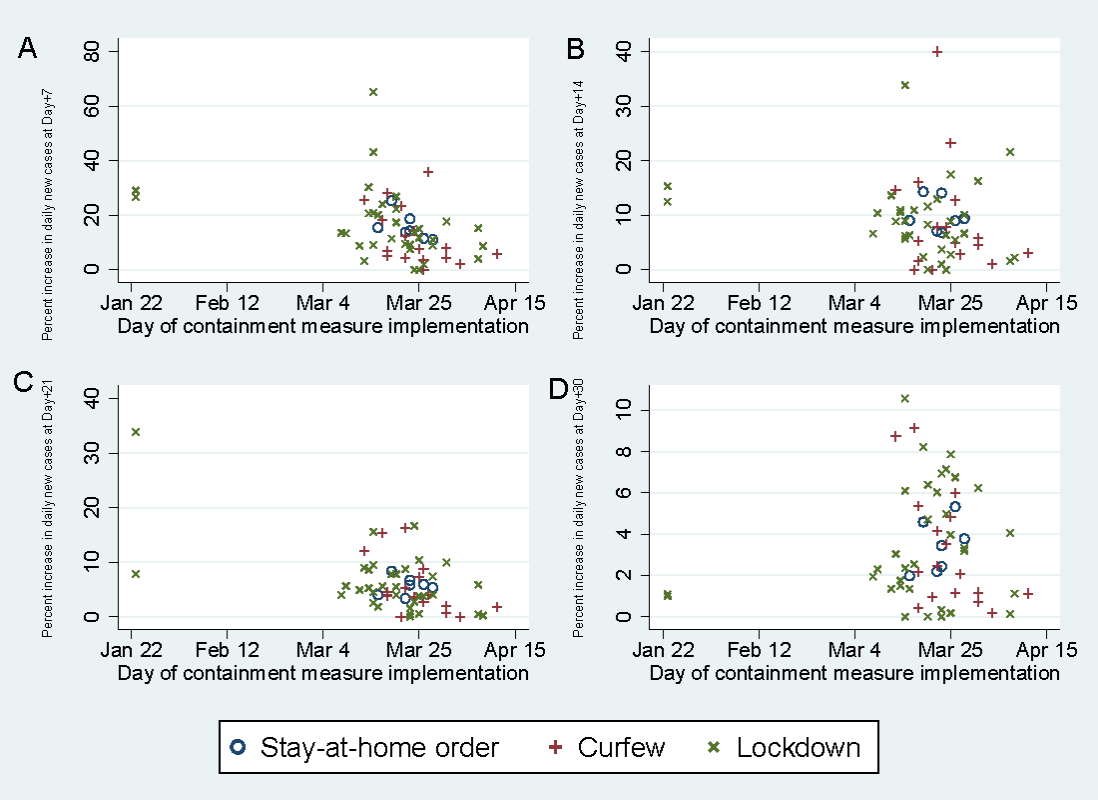
Percent increases in daily new cases at day +7 (A), day +14 (B), day +21 (C), and day +30 (D) vs start date of containment measure by type of containment measure.

Among countries with an average percent increase in daily new cases above 10 over seven days prior to the commencement of containment measures, a decreasing trend in percent increase in daily new cases was identified following the implementation of a stay-at-home order, curfew, or lockdown (Figure 5). mong the countries studied, 20% (n=11) had an average increase in daily new cases of 30-49% a week before intervention, while 13% (n=7) had an average increase in daily new cases of ≥50%. For countries with an average percent increase in daily new cases between 30-49%, 15 and 23 days were required to reduce the percent increase in daily new cases to 10 and 5, respectively. The distributions of percent increases of daily new cases at day +7, day +14, day +21 and day +30 by average percent increase before the commencement of containment measures in countries and epicenters are shown in Figure 6. A majority of countries (n=45, 83%) experienced experienced a lower percent increase in daily new cases at day +7 than their respective average percent increases before the commencement of measures, whereas only 2 countries had a higher percent increase in daily new cases at both day +14 and day +21 than that prior to containment intervention. The percent increases in daily new cases at day +7, day +14, day +21, and day +30 for each type of containment measure were similar regardless of the start date of the stay-at-home order, curfew, or lockdown (Figure 7).

**Figure 8:**
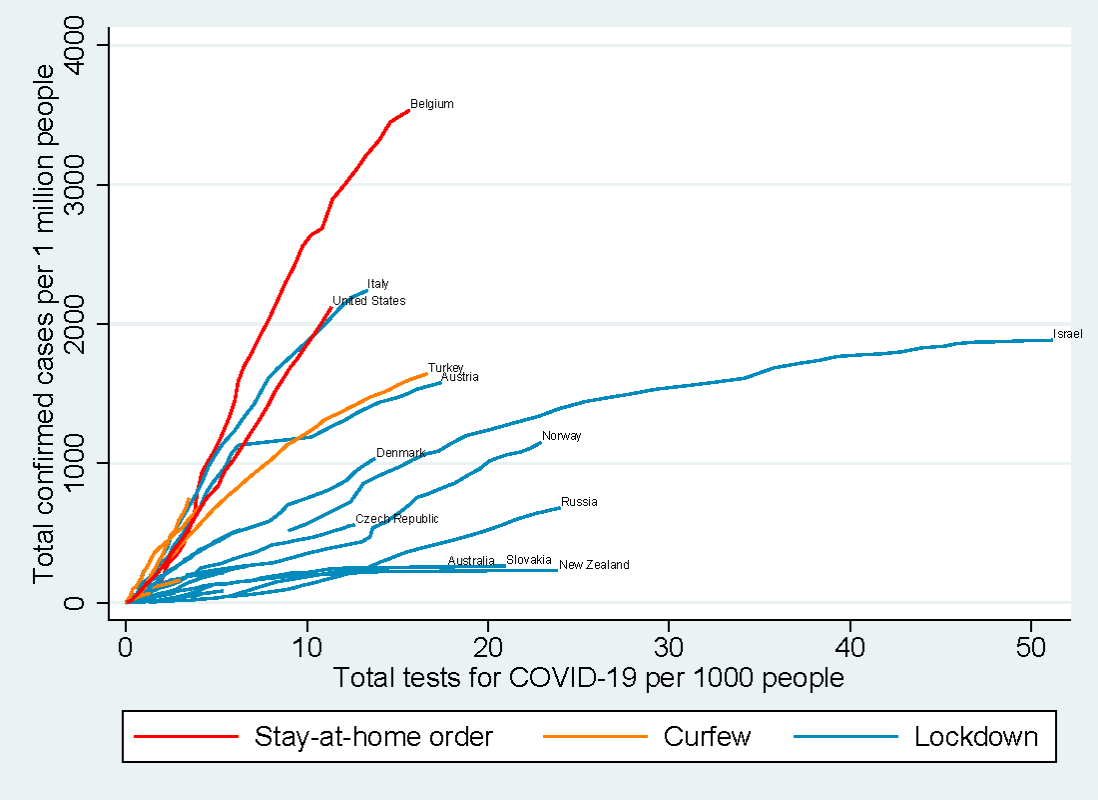
Total confirmed cases per 1 million people vs total tests for COVID-19 per thousand people in 54 countries by type of containment measure. COVID-19: coronavirus disease.

With respect to the type of containment measures, Figure 8 illustrates the relationship between the number of COVID-19 tests performed per 1000 people and the number of confirmed cases per 1 million people as reported by the health authority of each country. Countries that initiated lockdown demonstrated flattened curves, implying a reduction of the number of new confirmed cases per 1 million people, while these countries greatly expanded their scope of PCR testing. Countries adopting stay-at-home orders or curfews demonstrated a linear relationship between the number of COVID-19 tests performed and the number of confirmed cases. Increasing the number of COVID-19 tests performed had adjunctive effects on curbing the increase in daily new cases among locked-down countries.

## Discussion

### Principal Findings

Our findings suggest that different national containment measures, namely stay-at-home orders, curfews, and lockdowns, are associated with a decrease in the percent increase in daily new cases of confirmed COVID-19 infection. This is consistent with our previous understanding that the virus can be transmitted through respiratory droplets; hence, reducing social contact and population movement may help reduce the spread of this infectious disease. Among countries adopting a lockdown approach, increasing the number of COVID-19 tests may have had adjunctive effects on curbing the increase of confirmed daily new cases. The effects of stay-at-home orders, as described in this study, were contributed by the United States, the United Kingdom, Belgium, Germany, Ireland, and Japan. However, the effects of these orders were likely underestimated on a global scale, given that residents in many regions and countries (such as South Korea [21], Vietnam, Taiwan [22], and Hong Kong [23]) were strongly advised to stay home rather than being ordered to do so, which also appears to have been effective in reducing disease transmission [24]. Lockdown serves to prevent movement of infected individuals between regions. Therefore, lockdown was implemented in epicenters as an altruistic measure to contain the epidemic. While lockdown can cause severe disruption of international interactions, especially in this globalized era, further research is warranted to investigate whether entry screening and quarantine would achieve similar outcomes to those of lockdown. It is our view that stay-at-home orders are adopted to minimize community transmission, and lockdowns (or border restrictions) aim to reduce the number of imported cases. Curfew, however, only limits physical contact among individuals during specific time periods; this may lead to a less observable effect, if any.

In the United States, the most prominent country in North America, there was no single effective date of a stay-at-home order at the country level, as containment measures and government responses to the COVID-19 pandemic varied from state to state (ranging from March 19 to April 11). California was the earliest state to enact a stay-at-home order, on March 19, 2020; this was assumed to be the effective date of national containment measures for the United States in this study. Most states in the United States subsequently imposed stay-at-home orders, leading to a time lag between the effective date and actual disease control. It can be speculated that the delay in commencing these containment measures across the country contributed to the spike in percent increase in daily new cases with stay-at-home orders for the first few days immediately following the first effective date of implementation, as the United States accounted for almost half the percent increase in daily new cases during the peak observed.

A persistently low percent increase in daily new cases was observed in Asia throughout the study period. An upsurge of the number of COVID-19 cases alerted the health authorities in Asian countries and their residents during the early phase of the pandemic. With reference to the lessons learned during the SARS outbreak in 2003, Asian communities adopted various nonpharmaceutical measures to minimize disease transmission even before the announcement of health policies by local governments. This is evident in a report suggesting that nonpharmaceutical measures (such as hand hygiene, use of face masks, respiratory etiquette, and social distancing) have been incorporated into the local culture of Asian regions [25]. Countries in South America, however, showed signs of potential resurgence or a second wave of COVID-19 cases at the end of the study period. It is very concerning that containment measures did not appear to be effective in flattening the epidemic curve for individual countries in the region or that the health literacy of the public regarding COVID-19 infection should be improved. These concerns appear to be valid in light of the several subsequent spikes of percent increase in daily new cases identified across several South American countries (such as Argentina, Colombia, and Peru) following the lifting of containment measures before the disease was brought under control.

In addition to substantially lowering the percent increase in daily new cases in countries and regions with severe surges (≥100%), the investigated containment measures could also help less prevalent areas (<10%) to maintain a stable low percent increase in daily new cases. Accordingly, these interventions can be initiated at an early stage to flatten the curve and protect local health services from overwhelming demand. In view of the considerable economic cost associated with restriction of population movement, early public health interventions may also help alleviate the impact of prolonged recession and massive disruption of economic activities posed by mounting crises of newly confirmed cases and fatalities.

In this study, the relationships between the number of COVID-19 tests performed per 1000 people and the number of confirmed cases per 1 million people were examined by country and type of containment measures. These relationships were more obvious when the number of confirmed cases per million people was relatively small (around 1000 confirmed cases per 1 million people), which indicates the importance of leadership and coordination by local governments to make rapid expansion of PCR testing feasible and achievable in areas affected by the COVID-19 pandemic. Once the number of confirmed patients exceeded 1000, the effect of the same testing-to-population ratio almost disappeared; this may have occurred because health care facilities were overwhelmed by symptomatic patients, leaving no capacity to contact potential cases and conduct tests to identify an even larger number of carriers. Additionally, a country may fail to expand its testing capacity if the availability of testing agents becomes a rate-determining factor. Meanwhile, adoption of curfews and stay-at-home orders in countries did not appear to control the rise of new confirmed cases; hence, it can be postulated that testing must be coupled with other nonpharmaceutical public health control measures, such as quarantine and isolation, to exert effects on disease control.

The COVID-19 pandemic revealed how wealth inequity is associated with differential outcomes among countries. In our study, it was observed that high-income countries achieved a greater reduction in percent increase in daily new cases following the implementation of a stay-at-home order, curfew, or lockdown. This decline was more gradual among low-income and lower-middle-income countries, potentially because the crowded living conditions in these countries pose inherent risk of disease transmission. Moreover, other public health measures such as quarantine of exposed individuals may not be as effective in these countries because low-income employees who rely on their wages to meet financial obligations may avoid COVID-19 testing and subsequent forced quarantine. Therefore, development of a comprehensive public health intervention should be part of the strategic plan to manage the outbreak of infectious diseases alongside the promotion of health literacy of individuals, particularly in low-income countries and among people of lower social classes.

### Limitations

The current study has several limitations. First, in an attempt to control the disease outbreak, many governments implemented multiple public health interventions simultaneously or within a short timeframe [26]; thus, individual strategies could not be evaluated independently. Second, the effects of different containment measures on disease transmission can vary depending on the patterns of social contact at home, schools, and workplaces, which would likely be influenced by local cultures and the enforcement of public regulations. Third, the observational design of this study precluded causal inference. However, clinical trials are neither feasible nor ethical during the current public health emergency, and compelling evidence comparing the experience of one outbreak area with another in which different policies were adopted is still lacking.

During the pandemic, crowdsourced data collection has played an increasingly important role in providing timely and accurate data for disease surveillance [27]. A number of local crowdsourcing platforms, such as *Ushahidi* [28], have been working together to deliver real-time reports to the public to raise public awareness and facilitate the development of local contingency measures. This collaboration represents a potential advancement in the publication of timely epidemiological analyses and reports; however, the accuracy, ethics, and confidentiality of research data should not be undermined [29].

### Conclusion

National containment measures are essential to controlling the COVID-19 pandemic. In this study using crowdsourced data, countries that implemented stay-at-home orders, curfews, and lockdowns managed to curb the percent increase in daily new cases within a month; however, a resurgence in cases was observed in several South American countries two weeks after the commencement of containment measures. While no vaccine or effective treatment is yet available, the findings of our study can shed light on the impact of different containment measures and the priority of their implementation during this pandemic.

## Appendix

Multimedia Appendix 1 Income group and date of stay-at-home order, curfew and lockdown by countries.

## Abbreviations

COVID-19: coronavirus disease
MERS: Middle East respiratory syndrome
PCR: polymerase chain reaction
R0: initial transmission rate
Rt: effective transmission rate
SARS: severe acute respiratory syndrome
SARS-CoV-2: severe acute respiratory syndrome coronavirus 2
SURGE: Systematic Urgent Review Group Effort
