# Supplementary material for: Impact of National Containment Measures on Decelerating the Increase in Daily New Cases of COVID-19 in 54 Countries and 4 Epicenters of the Pandemic: Comparative Observational Study
Source: J Med Internet Res. 2020 Jul 22;22(7):e19904. doi: 10.2196/19904 (PMC7377680; doi:10.2196/19904)
Supplement: Multimedia Appendix 1 [file jmir_v22i7e19904_app1.docx]

Appendix. Income group and date of stay-at-home order, curfew and lockdown by countries

| **Country** | Continents | World Bank Income Classification | Stay-at-home date | Curfew date | Lockdown date | **References** |
| --- | --- | --- | --- | --- | --- | --- |
| 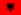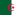  Algeria | Africa | Upper-middle-income | NA | 23-Mar-20 | 23-Mar-20 | http://covid19.sante.gov.dz/carte/ |
| 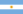  Argentina | South America | Upper-middle-income | NA | NA | 20-Mar-20 | https://www.argentina.gob.ar/coronavirus/informe-diario |
| 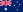  Australia | Australia | High-income | NA | NA | 23-Mar-20 | https://www.health.gov.au/news/health-alerts/novel-coronavirus-2019-ncov-health-alert/coronavirus-covid-19-current-situation-and-case-numbers |
| 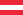  Austria | Europe | High-income | NA | NA | 16-Mar-20 | https://www.sozialministerium.at/Informationen-zum-Coronavirus/Neuartiges-Coronavirus-(2019-nCov).html |
| 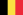  Belgium | Europe | High-income | 23-Mar-20 | NA | NA | https://epidemio.wiv-isp.be/ID/Pages/2019-nCoV_epidemiological_situation.aspx |
| 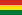  Bolivia | South America | Lower-middle income | NA | NA | 22-Mar-20 | https://www.minsalud.gob.bo/ |
| 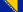  Bosnia and Herzegovina | Europe | Upper-middle-income | NA | 22-Mar-20 | NA | https://www.zzjzfbih.ba/dnevni-izvjestaj-za-potvrdene-slucajeve-covid-19-u-fbih |
| 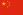  China | Asia | Upper-middle-income | NA | NA | 23-Jan-20 | <https://www.hubei.gov.cn/zwgk/hbyw/hbywqb/202001/t20200123_2014354.shtml> (lock down news)  <http://www.nhc.gov.cn/xcs/yqtb/list_gzbd.shtml> (official info website) |
| 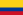  Colombia | South America | Upper-middle-income | NA | NA | 24-Mar-20 | https://d2jsqrio60m94k.cloudfront.net/ |
| 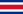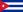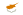  Cyprus | Europe | High-income | NA | 31-Mar-20 | NA | https://www.pio.gov.cy/coronavirus/ |
| 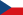  Czech Republic | Europe | High-income | NA | NA | 14-Mar-20 | https://www.vlada.cz/en/media-centrum/aktualne/measures-adopted-by-the-czech-government-against-coronavirus-180545/ |
| 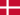  Denmark | Europe | High-income | NA | NA | 13-Mar-20 | https://www.ssi.dk/aktuelt/sygdomsudbrud/coronavirus |
| 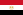  Egypt | Africa | Lower-middle income | NA | 25-Mar-20 | NA | https://sis.gov.eg/Story/144148?lang=en-us |
| 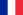  France | Europe | High-income | NA | NA | 17-Mar-20 | https://www.santepubliquefrance.fr/maladies-et-traumatismes/maladies-et-infections-respiratoires/infection-a-coronavirus/articles/infection-au-nouveau-coronavirus-sars-cov-2-covid-19-france-et-monde |
| 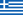  Greece | Europe | High-income | NA | NA | 23-Mar-20 | https://www.moh.gov.gr/articles/ministry/grafeio-typoy/press-releases/6866-dhlwsh-ekproswpoy-toy-ypoyrgeioy-ygeias-gia-ton-neo-koronoio-kathhghth-swthrh-tsiodra-13-3-2020 |
| 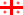  Georgia | Europe | Upper-middle-income | NA | 31-Mar-20 | 31-Mar-20 | https://agenda.ge/en/news/2020/980 |
| 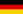  Germany | Europe | High-income | 16-Mar-20 (Gathering Ban) | NA | 23-Mar-20 | https://www.rki.de/DE/Content/InfAZ/N/Neuartiges_Coronavirus/Fallzahlen.html |
| 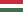  Hungary | Europe | High-income | NA | NA | 28-Mar-20 | https://koronavirus.gov.hu/ |
| 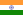  India | Asia | Lower-middle income | NA | NA | 25-Mar-20 | https://www.mohfw.gov.in/ |
| 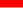  Indonesia | Asia | Lower-middle income | NA | 17-Apr-20  (Pekanbarn) | 10-Apr-20 (Jarkata) | https://experience.arcgis.com/experience/57237ebe9c5b4b1caa1b93e79c920338 |
| 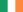  Ireland | Europe | High-income | 28-Mar-20 | NA | NA | https://www.gov.ie/en/news/7e0924-latest-updates-on-covid-19-coronavirus/ |
| 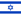  Israel | Asia | High-income | NA | NA | 2-Apr-20 | https://govextra.gov.il/ministry-of-health/corona/corona-virus-en/ |
| 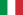  Italy | Europe | High-income | NA | NA | 9-Mar-20 | <http://www.salute.gov.it/portale/home.html>  http://www.trovanorme.salute.gov.it/norme/dettaglioAtto?id=73629 |
| 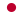 Japan | Asia | High-income | 26-Mar-20 | NA | NA | https://www.metro.tokyo.lg.jp/tosei/governor/governor/kishakaiken/2020/03/27.html |
| 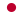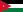  Jordan | Asia | Upper-middle-income | NA | 21-Mar-20 | NA | https://corona.moh.gov.jo/ar |
| 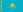  Kazakhstan | Asia | Upper-middle-income | NA | NA | 19-Mar-20 | http://www.gov.kz/memleket/entities/dsm/press?lang=en |
| 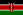  Kenya | Africa | Lower-middle income | NA | 27-Mar-20 | NA | <https://www.health.go.ke/> |
| 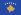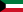  Kuwait | Asia | High-income | NA | 22-Mar-20 | NA | https://corona.e.gov.kw/ |
| 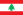  Lebanon | Asia | Upper-middle-income | NA | 26-Mar-20 | NA | https://www.moph.gov.lb |
| 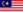Malaysia | Asia | Upper-middle-income | NA | 18-Mar-20 | NA | http://www.moh.gov.my/index.php/pages/view/2019-ncov-wuhan |
| 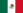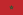  Morocco | Africa | Lower-middle income | NA | NA | 20-Mar-20 | http://www.covidmaroc.ma/ |
| 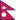  Nepal | Asia | Low-income | NA | NA | 24-Mar-20 | https://heoc.mohp.gov.np/ |
| 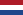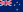  New Zealand | Australia | High-income | NA | NA | 25-Mar-20 | https://www.health.govt.nz/our-work/diseases-and-conditions/covid-19-novel-coronavirus/covid-19-current-cases |
| 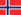  Norway | Europe | High-income | NA | NA | 12-Mar-20 | https://www.fhi.no/sv/smittsomme-sykdommer/corona/dags--og-ukerapporter/dags--og-ukerapporter-om-koronavirus/ |
| 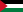  Palestine | Asia | NA | NA | NA | 23-Mar-20 | http://english.pnn.ps/2020/03/22/palestine-pm-bans-movement-between-and-inside-of-cities/ |
| 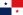  Panama | South America | High-income | NA | 13-Mar-20 | NA | <https://www.embassyofpanama.org/covid-19> |
| 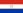  Paraguay | South America | Upper-middle-income | NA | NA | 20-Mar-20 | https://www.mspbs.gov.py/reportes-covid19.html |
| 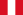  Peru | South America | Upper-middle-income | NA | 18-Mar-20 | 15-Mar-20 | <https://www.gob.pe/8371-que-son-los-coronavirus/pages> |
| 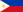  Philippines | Asia | Lower-middle income | NA | NA | 15-Mar-20 | https://www.who.int/philippines/emergencies/covid-19-in-the-philippines/covid-19-sitreps-philippines  <https://www.gov.ph/> |
| 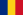  Romania | Europe | Upper-middle-income | NA | NA | 25-Mar-20 | <http://www.ms.ro/> |
| 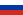  Russia | Europe | NA | NA | NA | 31-Mar-20 | https://www.rosminzdrav.ru/ministry/covid19 |
| 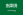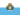  Saudi Arabia | Asia | High-income | NA | NA | 9 -Mar-20  (Qatif) | <https://www.moh.gov.sa/en/Pages/Default.aspx> |
| 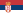  Serbia | Europe | Upper-middle-income | NA | 17-Mar-20 | 15-Mar-20 | https://covid19.rs/ |
| 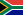  South Africa | Africa | Upper-middle-income | NA | NA | 26-Mar-20 | <http://www.health.gov.za/index.php/outbreaks/145-corona-virus-outbreak/465-corona-virus-outbreak> |
| 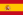  Spain | Europe | High-income | NA | NA | 14-Mar-20 | https://www.mscbs.gob.es/profesionales/saludPublica/ccayes/alertasActual/nCov-China/situacionActual.htm |
| 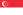  Singapore | Asia | High-income | NA | NA | 7-Apr-20 | <https://www.moh.gov.sg/> |
| 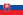  Slovakia | Europe | NA | NA | NA | 8-Apr-20 | https://www.health.gov.sk/Clanok?koronavirus-pripady-dalsie |
| 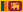  Sri Lanka | Asia | Upper-middle-income | NA | 18-Mar-20 | NA | https://hpb.health.gov.lk/en/api-documentation |
| 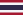  Thailand | Asia | Upper-middle-income | NA | 3-Apr-20 | 25-Mar-20 | <https://ddc.moph.go.th/viralpneumonia/eng/index.php> |
| 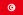  Tunisia | Africa | Lower-middle income | NA | 18-Mar-20 | NA | https://covid-19.tn |
| 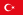  Turkey | Europe | Upper-middle-income | NA | 11-Apr-20 | NA | https://covid19.saglik.gov.tr/ |
| 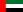  United Arab Emirates | Asia | High-income | NA | 26-Mar-20 | NA | https://wam.ae/en/archive?q=COVID |
| 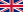  United Kingdom | Europe | High-income | 23-Mar-20 | NA | NA | https://www.gov.uk/government/publications/covid-19-track-coronavirus-cases |
| 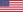  United States | North America | High-income | 19-Mar-20 | NA | NA | https://www.cdc.gov/coronavirus/2019-ncov/cases-in-us.html |

| **Epicenter** | Continents | Income | Stay-at-home date | Curfew date | Lockdown date | **References** |
| --- | --- | --- | --- | --- | --- | --- |
| China - Wuhan | Asia | Upper-middle-income | NA | NA | 23-Jan-20 | https://www.hubei.gov.cn/zwgk/hbyw/hbywqb/202001/t20200123_2014354.shtml |
| United States - New York | North America | High-income | 22-Mar-20 | NA | NA | https://coronavirus.health.ny.gov/home |
| Italy - Lombardy | Europe | High-income | NA | NA | 22-Feb-20 | https://www.comune.milano.it/documents/20126/3681874/Coronavirus.+Ordinanza+Ministero+della+Salute+e+Regione+Lombardia.pdf/59a5848d-bec4-72aa-bb50-4dda7c8df91d?t=1582528914878 |
| Spain - Madrid | Europe | High-income | NA | NA | 15-Mar-20 | https://www.boe.es/buscar/pdf/2020/BOE-A-2020-3692-consolidado.pdf |

NA = Not Applicable
